# Manually-parcellated gyral data accounting for all known anatomical variability

**DOI:** 10.1038/sdata.2019.1

**Published:** 2019-01-29

**Authors:** Shadia S. Mikhael, Grant Mair, Maria Valdes-Hernandez, Corné Hoogendoorn, Joanna M. Wardlaw, Mark E. Bastin, Cyril Pernet

**Affiliations:** 1 University of Edinburgh, Centre for Clinical Brain Sciences (CCBS), The Chancellor’s Building, 49 Little France Crescent, Edinburgh EH16 4SB, UK; 2 Canon Medical Research Europe, Bonnington Bond, 2 Anderson Place, Edinburgh EH6 5NP, UK

**Keywords:** Cognitive neuroscience, Cognitive ageing, Neurology

## Abstract

Morphometric brain changes occur throughout the lifetime and are often investigated to understand healthy ageing and disease, to identify novel biomarkers, and to classify patient groups. Yet, to accurately characterise such changes, an accurate parcellation of the brain must be achieved. Here, we present a manually-parcellated dataset of the superior frontal, the supramarginal, and the cingulate gyri of 10 healthy middle-aged subjects along with a fully detailed protocol based on two anatomical atlases. Gyral parcels were hand-drawn then reviewed by specialists blinded from the protocol to ensure consistency. Importantly, we follow a procedure that allows accounting for anatomical variability beyond what is usually achieved by standard analysis packages and avoids mutually referring to neighbouring gyri when defining gyral edges. We also provide grey matter thickness, grey matter volume, and white matter surface area information for each parcel. This dataset and corresponding measurements are useful in assessing the accuracy of equivalent parcels and metrics generated by image analysis tools and their impact on morphometric studies.

## Background & Summary

To study large morphological brain changes associated with ageing and disease, magnetic resonance imaging (MRI) data of the brain are acquired then analysed, often by segmenting tissue types (i.e. grey matter, white mater, cerebro-spinal fluid) and parcellating the brain into regions which are defined by gyri and sulci. Most gyral volumes that are publicly available have been derived either automatically or semi-automatically. The latter group typically stems from automated parcellation (or segmentation), followed by manual changes, correcting for errors often due to inaccuracies in the segmentation and/or the parcellation scheme^1,2^. Because of the manual aspect, they are usually classified as ‘manually-generated’. These datasets are considered as the ‘gold standard’ and exist for the various brain regions— cortical^2,3^, subcortical^4^, abnormal (such as tumours or lesions^5^), and the whole brain (LPBA40^6^; NeuAtlas (Neuromorphometrics, Inc. NeuAtlas, http://www.neuromorphometrics.com/?p=315)— although they are not always available to the public^4^. In theory however, manually-generated data should only refer to volumes that are hand-drawn, from beginning to end, and are therefore free of software-related bias. Because the manual process is very tedious and time-consuming, such datasets are very rare (e.g., MNI-HISUB25^7^).

Irrespective of the nature of segmentation, available datasets rarely provide population demographics, details on how the regions have been drawn or obtained, or whether anatomical variations are considered^1^. Scarce attempts have been made in retrospect to address these issues^2^. This situation proves to be challenging for end-users following such tools’ parcellation protocols, particularly when it comes to anatomical variability, leaving lots of room for assumptions, misinterpretations, and inconsistent parcellations, all of which are undesirable. Previous work suggests that automated image analysis tools reliant on these protocols go on to produce differing representations of the similarly named same gyrus, rendering interpretation and/or comparisons impossible^1,8,9^.

For more clarity on these essential aspects, we created a new parcellation protocol^10^, based on two anatomical atlases^11,12^. The protocol describes 3 particular gyri— the superior frontal gyrus (SFG), the supramarginal gyrus (SMG), and the cingulate gyrus (CG)— while providing methodical step-by-step instructions for identifying each of their borders. Ultimately, all gyri should be defined in a manner that allows for morphological variability to be accounted for, making automated parcellation tools more reliable. Here we focused on these 3 gyri as they are known to exhibit structural changes in ageing^13^ and dementia^14–17^ populations, and to significantly differ between sexes^13,18^.

We provide parcellation of 10 subjects’ left and right SFG, SMG, and CG, which should prove useful to the brain imaging community given how rare manually-segmented datasets are. Although the sample size is small, these data are a good example of the morphological changes that an upper middle-aged healthy population undergoes, and a valuable ground truth for studies investigating the effects of ageing and/or disease on cohorts of similar age. We include complete population demographics and a detailed protocol that accounts for anatomical variability such as interruptions, connections and branching. This 60-gyrus dataset could also be used as a reference (rather than absolute truth) to assess inter-package parcellation differences or to validate a novel or improved parcellation tool, independently of the borders defined in the protocol, because the manually parcellated data allow checking for the anatomical variability we describe.

## Methods

### Dataset Methods

#### Subjects

Ten healthy right-handed non-smoking subjects (5 male, 5 female, age range 55–64 years old), not on any medication, were randomly selected among a larger NIH-funded study (NIH grant R01 EB004155) involving 80 healthy subjects. MRI data were collected at the Western General Hospital (Edinburgh, UK) and structural scans were examined by a fully-qualified radiologist, confirming all subjects were in good health.

#### Data acquisition

The scans and cognitive tests were acquired and administered in 2008–2012, (data summarized in Table 1) prior to the development of community reporting standards, however, all data were systematically collected and reported. The local ethics committee approved the study and informed consent was obtained from each patient. For each of the 10 subjects in this dataset, 4 MRI volumes were obtained: coronal high resolution 3D T1-weighted (T1w), axial T2-weighted (T2w), T2*-weighted and T2 FLAIR. All scans were acquired on a 1.5 T MRI scanner (General Electric, Milwaukee, WI, USA) at the Brain Research Imaging Centre in Edinburgh (UK). Further details can be found in Tables 2 and 3 of the Data Records section).

A medical questionnaire and a battery of cognitive subtests from the 4^th^ edition of the Wechsler Adult Intelligence Scale (WAIS-IV^19^) were administered to each healthy volunteer. Checks were made to ensure that they scored within the normal range (Table 1).

The general practitioner (GP) of every volunteer was contacted twice throughout the study: once to inform them of the subject’s participation in the study’s details (along with the study’s information sheet), and once more to inform them of the scan’s outcome.

#### Data preparation

**Figure 1:**
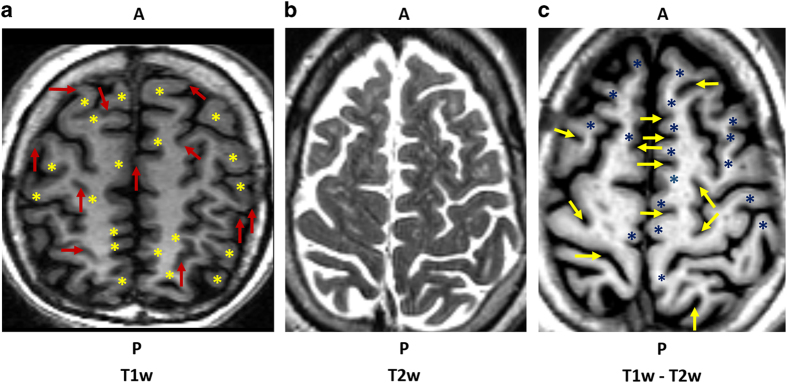
Contrast enhancement using T1w-T2w volume difference (subject 2). The raw T1w volume (**a**) had low GM-WM contrast (yellow stars) as well as low GM-CSF contrast (red arrows), particularly visible at the sharp bends in the cortical surface and the small CSF spaces. By registering the subject’s T1w volume to the T2w volume (**b**), bias field correction, and subtraction, we generated a difference volume (**c**) with enhanced contrast (blue stars and yellow arrows), allowing for simpler and more accurate manual parcellation.

Given the limited contrast between grey matter (GM), white matter (WM) and cerebrospinal fluid (CSF) at 1.5 T, we adopted a multispectral method to enhance the raw subject volumes prior to parcellation (Fig. 1). The method would better the anatomical accuracy without altering the intensities in the neighbourhood of the tissue boundaries. To achieve this we combined the T1-weighted and T2-weighted volumes as detailed in the steps below:

1. convert all volumes from dicom to ANALYZE 7.5 format (.hdr and .img files) using in-house software as we had initially intended to parcellate in Analyze 12 (Analyze12. *AnalyzeDirect, Inc.* https://analyzedirect.com/)
2. convert the coronal T1w volume to an axial T1w volume
3. flip the T1w volume, using Analyze 12, along the y- and z-axes for neurological orientation
4. register the T1w volume to the T2w volume using FLIRT^20–22^. The T1w and T2w volumes are now in radiological convention
5. flip the T1w and T2w volumes along the y-axis for correct neurological orientation using Analyze 12
6. bias field correction of the co-registered T1w volume in 3D Slicer (http://www.slicer.org), version 4.3.1^23^, using the ‘N4ITK MRI bias correction’ module and default N4 parameters. The T1w volume is saved again in radiological convention
7. flip the T1w and T2w volumes again along the y-axis for neurological correct orientation using Analyze 12
8. exclude the least occurring intensities in the T1w volume, as shown in the histogram, using Analyze 12
9. subtract the T2w volume from the T1w volume using Analyze 12’s image calculator

### Parcellation Method

The variability of folding patterns is very large, making it a challenge to accurately incorporate them into gyral definitions. The number of folds in a gyrus may increase or decrease, and sulci may experience a combination of branching, connections, interruptions, and absences^11^. Given that sulci are the landmarks most commonly used for defining gyral borders, their misrepresentation can significantly skew gyral representations, producing false over- or under-estimations of them. It therefore becomes crucial to define parcellation protocols in a manner that is clear and flexible enough to incorporate and reflect all recognised variability, but with consistent reproducibility. Existing protocols fail to do so as they either omit some forms of variability reported in the literature or fail to clarify how a particular form (e.g., sulcal absence, sulcal discontinuity, double sulcal occurrence, etc.) shall be addressed^1^.

#### Protocol details

Gyral parcellation is most often equivalent to the parcellation of GM, which is what we endorse in the present parcellation protocol^10^. Cortical GM is bound by CSF externally and WM internally. To define these two borders, or surfaces, we instruct the user to create two separate paired masks (or borders), one for the GM’s outer border and one for its inner border.

We consulted 2 brain atlases to devise a comprehensive protocol for the SFG, SMG and CG. The first atlas, by Duvernoy^12^, indicates the general location of each gyrus, in various views and throughout the brain, however, sulcal variability details such as interruptions, connections, and branching are missing. The second, by Ono *et al*.^11^, thoroughly describes sulcal patterns and variability, but rarely in relation to adjacent gyri. Despite their variability, sulci are the main gyral delimiters, necessitating a clear understanding of them and of the consequent gyral variations. By combining the valuable details from both anatomical sources (gyral location from the first and the patterns and variability of their delimiters from the second), we moulded a single, accurate, consistent and detailed protocol for the three gyri. For each gyrus we first specify the view (axial, sagittal, or coronal) in which it is to be identified and drawn, while naming all gyral borders, mainly sulci. We then provide detailed, step-by-step instructions on drawing the gyrus from start to end, along the direction in which it propagates, in addition to information on the known variations that may be encountered and how to address them. We occasionally resort to a notch or artificial line rather than a sulcus to mark a clear start or end to the segmentation, for the sake of consistency and reproducibility. Illustrations accompany the instructions for clarification purposes.

Because of the gyral folding pattern (frequency and sharpness of turns), software can fail to accurately outline the anatomy (e.g., sulcus, gyrus, or surface). Furthermore, with cortical thickness ranging from 1 mm to ~5 mm^24,25^, it becomes more difficult to identify the grey and white matter boundaries due to partial volume effects. We therefore used multiple segments to represent these boundaries, while accurately following each rise and fall in the cortical surface.

#### Parcellation details

**Figure 2:**
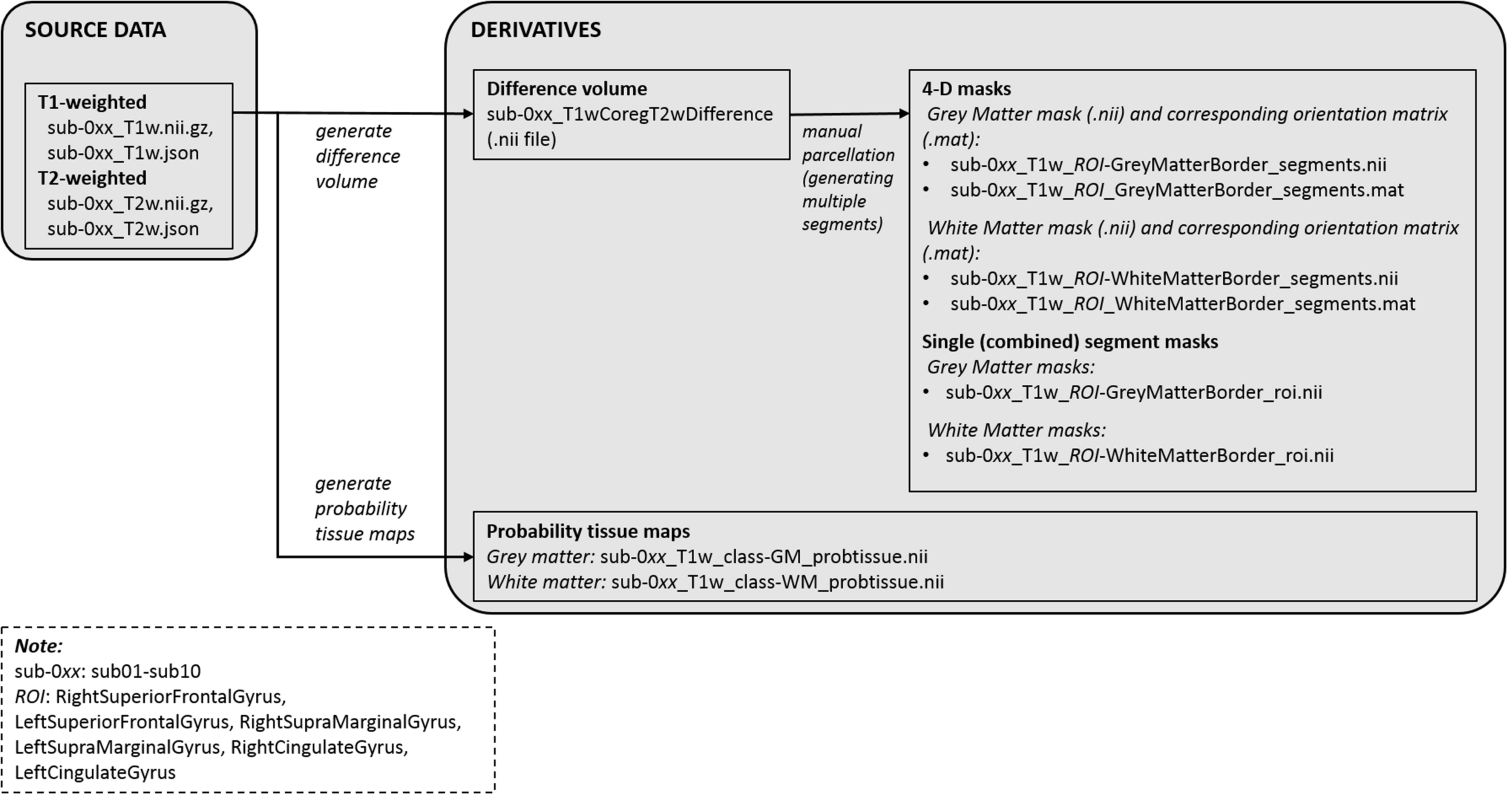
Workflow depicting the stages followed to create the derivative masks. From the source T1w and T2w data, difference volumes were generated and gyral borders were outlined using multiple segments. The segments were then combined into a single 4D and a single 3D file for each gyrus (top). For comparison purpose, a multispectral segmentation of grey and white matter tissue was also performed using SPM12 (bottom).

For each of the 10 subjects, we first loaded the enhanced difference volume (T1w-T2w) in MRIcron (https://www.nitrc.org/projects/mricron, version 22DEC2015), of voxel size 1 × 1 × 2 mm. Then we manually traced the paired masks representing outer and inner GM borders for the SFG, SMG and CG in both the right and left hemispheres following the pre-defined protocol^10^. Importantly, this was done for every discontinuity and every sharp change in curvature. As a result, many segments were required to outline a single gyrus. The segments were merged to form a single volume for each gyrus. A workflow detailing how the derived data were made is illustrated in Fig. 2. The workflow in Fig. 2 illustrates how the derived data were made. We identified a total of 66 sulcal (CS and SFS) discontinuities in the axial plane at the SFG, 24 gyral discontinuities in the sagittal plane at the SMG, and 103 sulcal (CS) discontinuities in the sagittal plane at the CG, all of which are a result of the folding nature of cortical gyri (Table 4). We also identified 6 double sulcal (CS), and therefore 6 double gyral (CG) occurrences (n = 6). These anatomical variations (sulcal discontinuities and double sulcal occurrences) will have influenced our gyral parcellations.

A standard approach for validating a parcellation protocol is to obtain equivalent manual segmentations from several experts. We instead sought to validate the consistency of our manual parcellation, i.e., the anatomical landmarks defining the gyral borders which are known to vary across hemispheres and subjects. This alternative method is very similar to that of Klein and Tourville^2^ where the authors first automatically parcellated the brains using their older protocol^26^, then followed it with manual corrections based on the anatomical variability they detected in each of the subjects which the automated method failed to identify. Here, after reviewing the 2 anatomical brain atlases^11,12^ and writing the protocol, the first author (SM) manually segmented and parcellated all 10 subjects’ regions of interest. They were then revised for landmark consistency and accuracy by 2 experts, CP and GM, who were blinded from the protocol. When inconsistency was found across subjects, the protocol was amended, and the regions were redrawn and reviewed again. Consecutive revisions by the 3 authors and protocol updates continued until an agreement was reached on the dataset’s anatomical accuracy and consistency as well as the comprehensiveness of the protocol with regards to variability.

Because we did not seek to validate the tissue segmentation itself (grey matter-cerebrospinal fluid and grey matter-white matter borders), but the consistency of the parcellation scheme, the signal intensity (after enhancement) and spatial resolution of 1.5 T MRI is adequate for this task.

### Code Availability

The Matlab (https://uk.mathworks.com/products/matlab.html, R2016a) code used to generate the combined segments as derivative files can be found alongside the data (Data Citation 1).

To validate the manual parcellation of the gyri of interest, we computed the mean thickness of each (GM_th_), using all paired segments, and compared them to FreeSurfer version 5.1’s outputs. This was done using the Masks2Metrics (M2M) software version 1.0^27,28^, freely available to all users under the GNU General Public License. The latest version of the software is available at https://github.com/Edinburgh-Imaging/Masks2Metrics.

## Data Records

All the data used and created by this study are available in the Edinburgh DataShare repository (Data Citation 1). Data are organized following the Brain Imaging Data Structure (BIDS^29^, also defined at http://bids.neuroimaging.io/) with the T1 and T2 weighted volumes as source and the parcellation volumes as derivatives. Tables 2 and 3 summarize the MRI parameters and Table 5 summarizes the additional ROI details. The number of paired segments ranged from 24 to 59 for the SFG, 3 to 16 for the SMG, and 16 to 54 for the CG. This number tended to increase with the increase in cortical folding as well as cortical variability such as discontinuities and double gyrus occurrences.

The grey matter thickness, grey matter volume, and white matter surface area information for each parcel is available in Data Citation 2 (SuperiorFrontalGyrus.tsv, SupraMarginalGyrus.tsv, and CingulateGyrus.tsv). Columns 2–10 contain FreeSurfer-derived metrics, columns 11–19 contain Masks2Metrics-derived metrics, and columns 20–22 contain mean modified Hausdorff distance (MMHD, https://uk.mathworks.com/matlabcentral/fileexchange/29968-modified-hausdorff-distance) metrics. Further metrics details can be found in the Technical Validation section.

## Technical Validation

As mentioned in the ‘Parcellation Details’ section, we validated the protocol using a blinded review of the data checking between subjects consistency whilst still considering of all known forms of cortical variability. A scanner strength of 1.5 T was sufficient as we did not seek to validate the grey matter-cerebrospinal fluid and grey matter-white matter border segmentation itself, but the consistency in parcellation and border identification despite all sorts of cortical variability, across hemispheres and subjects.

To quantitatively assess the data parcels, we compared the average thickness, volume and surface area of each gyrus/sulcus from our parcellation to that of FreeSurfer (version freesurfer-Linux-centos4_x86_64-stable-pub-v5.1.0). Although anatomical variations are not accounted for as much as in our protocol^1^, it still provides a valuable comparison, as variations should not be substantial, in particular for average thickness^30^. We also computed MMHD which, like FreeSurfer, is computed by averaging the shortest distance from each voxel on one segment to the other segment, in both directions. M2M on the other hand measures the perpendicular from one segment to the other, in both directions. We computed this distance for the inner and outer GM masks and compared it to those of the other two methods (Table 6).

We ran FreeSurfer using default settings to process each subject’s T1w NifTI volume, then limited our investigations to the output of the Desikan-Killiany protocol^26^ – specifically SFG, CG and SMG parcellations – and corresponding measurements. FreeSurfer developers recommend that manual checks and corrections typically follow automated parcellation, however, they were omitted in our case for two main reasons: (1) so as not to introduce human error/bias, and (2) the corrections would not have a drastic effect on ROI average thickness, while equalizing volume and surface area because of our edits (detailed under ‘Data Preparation’). For the SFG and SMG, metrics for the corresponding FreeSurfer labels were used, ‘superiorfrontal’ (label 80) and ‘supramarginal’ (label 83) respectively, while for the CG, 4 FreeSurfer labels (‘rostralanteriorcingulate’, ‘caudalanteriorcingulate’, ‘posteriorcingulate’ and ‘isthmuscingulate’, or labels 78,55,75,62) were used to ensure ‘like-for-like’ comparison.

**Figure 3:**
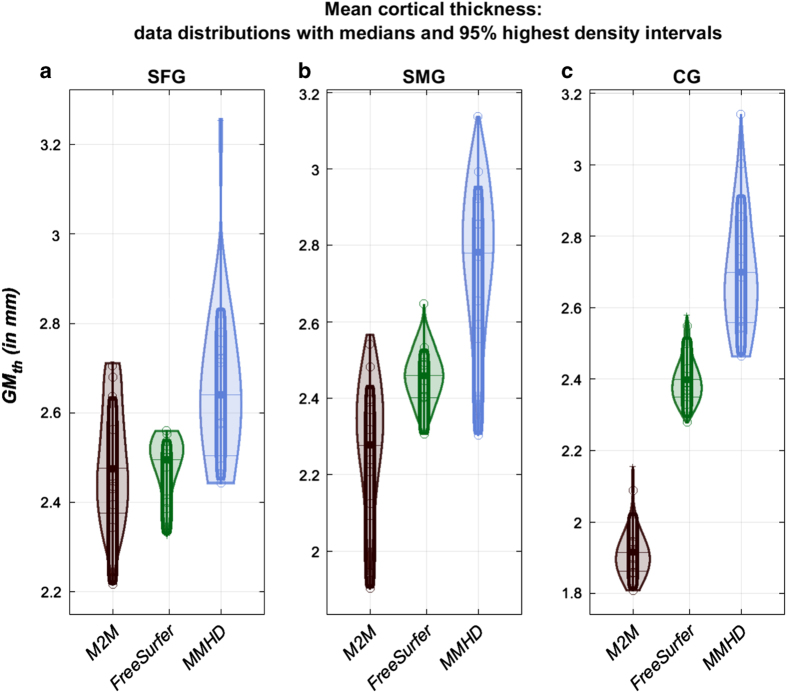
Cortical thickness measurements as calculated by Masks2Metrics, FreeSurfer and the mean modified Hausdorff distance (MMHD), along with corresponding non-parametric density estimates of the thickness (the thick lines represent the median). The regions measured by the tools, from left to right, are the SFG (**a**), SMG (**b**), and CG (**c**).

Generally, gyrus thicknesses, as derived by the 3 methods, are in agreement with lateral (3.5 mm), medial (2.7 mm) and overall (2.5 mm) cortical thicknesses measured in post-mortem brains^25^ (Fig. 3). A percentile bootstrap on median differences showed no difference for the SFG between M2M and FreeSurfer (median difference 0.02 [−0.08 0.13] p = 0.68), and lower M2M estimates for the SMG (median difference 0.18 [0.09 0.3] p = 0.001) and CG (0.51 [0.36 0.59] p = 0.001). These last two differences are mainly due to the large variability in the ROIs’ bordering landmarks. Furthermore, the CG consisted of a large number of short segments implying that fewer perpendicular M2M thickness measurements were nonzero compared to the corresponding shorter-distance measurements of FreeSurfer. Because both the MMHD and FreeSurfer seek the shortest distance, their results should be comparable. FreeSurfer measurements are however made in 3D while our approaches rely on 2D leading to an overestimation (SFG median difference 0.18 [0.06 0.27] p = 0.006; SMG median difference 0.31 [0.13 0.45] p = 0.001; CG median difference 0.28 [0.12 0.52] p = 0.002). Together these results indicate that accounting for variability by creating segments is essential so as to not over-estimate thickness.

**Figure 4:**
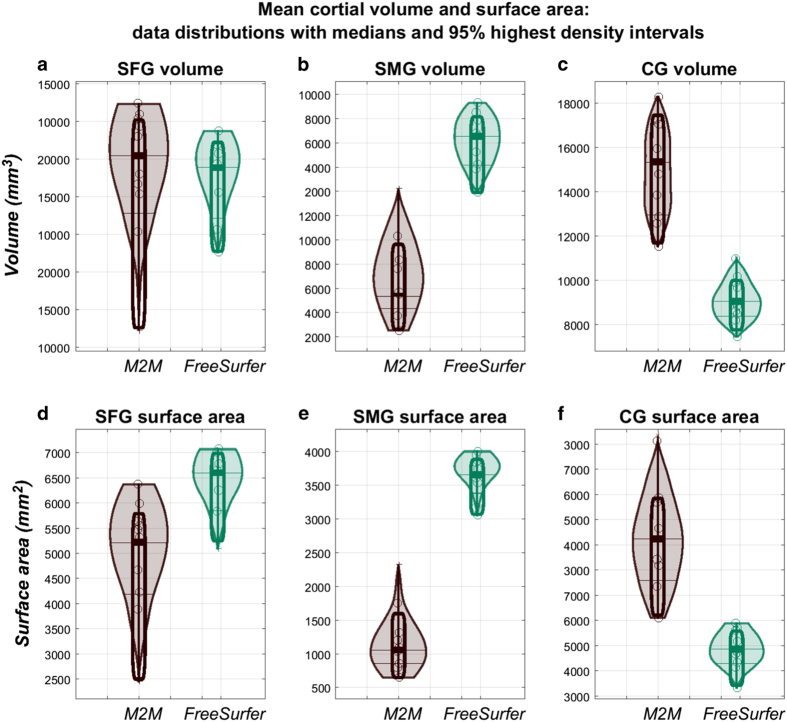
Volume (GM_vol_) and surface area (WM_sa_) measurements computed by M2M and FreeSurfer, along with their corresponding non-parametric density estimates (thick lines represent the median). SFG metrics (**a**,**d**) are most similar between the two techniques, although a larger metric distribution is seen at both the SFG (**a**,**d**) and CG (**c**,**f**), mainly due to cortical variability in the cingulate sulcus which is not always accounted for by FreeSurfer. The greatest disagreement between the methods is seen at the SMG where we observed smaller parcel volumes (**b**) and surface areas (**e**) with M2M than with FreeSurfer.

**Figure 5:**
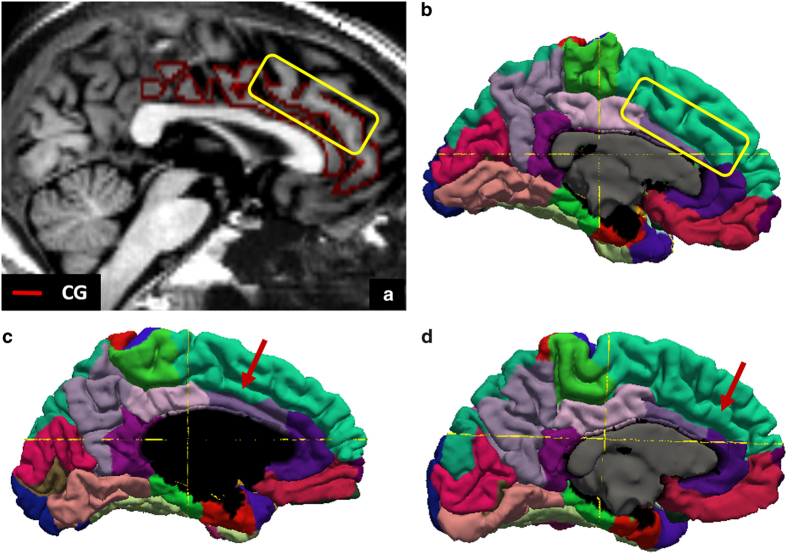
A demonstration of protocol differences stemming from cortical variability, as seen in several subjects. With our protocol, the double cingulate sulcus scenario for subject 5’s left hemisphere, shown in the T1w-T2w difference volume, contributes to a CG with two folds (**a**), whereas with FreeSurfer’s protocol, the superior fold is mostly (inside the yellow box) a part of the SFG (**b**). Parts of the upper CG fold are similarly omitted by FreeSurfer in the left hemispheres of subjects 1 (**c**) and 6 (**d**).

Since our data accounts for anatomical variations such as a double CG, which we encountered in 6 of the 10 subjects’ hemispheres, significantly larger mean CG GM volume (Fig. 4c) and in turn mean WM surface area (Fig. 4f) measurements are observed in the manually-derived parcels compared to their corresponding FreeSurfer counterparts (median volume difference 6304.2 [3627.08 8806.70] p = 0.002; median surface area difference 2193.34 [1276.27 3179.56] p = 0.002). In the event of a double CG, the SFG on the medial surface ‘loses’ its inferior-most fold to the CG in our protocol (Fig. 5a), compared to FreeSurfer (Fig. 5b–d). This explains why our manually-derived SFG WM surface areas are smaller than their corresponding FreeSurfer-derived ones ((Fig. 4d- median difference 1379.99 [529.01 2468.41] p = 0.002), and to a lesser extent also GM volumes, although not significantly (Fig. 4a - median volume difference 632.61 [−3105.74 3680.1] p = 0.63). The double cingulate occurrences in our cohort also explain the wider distributions for both the CG (Fig. 4c,f) and SFG (Fig. 4a,d).

The greatest disagreement between the two methods was evident when calculating SMG metrics. This is most likely due to the inferior border of the SMG in our protocol being more superior than that of FreeSurfer, lending to smaller manually-segmented parcels, and therefore smaller parcel volumes (Fig. 4b) and inner mask surface areas (Fig. 4e) with M2M than with FreeSurfer (median volume difference 6588.74 [4165.12 7873.76] p = 0.002; median surface area difference 2598.92 [2012.17 2954.39] p = 0.002).

Not only do parcellation schemes (and tools) have direct implications on the morphometrics of the regions they outline, but also on any concomitant analyses. To demonstrate this, we conducted a regression analysis on the three CG metrics (thickness, volume and surface area), as measured by M2M and FreeSurfer, with respect to the National Adult Reading Test (NART) score. The NART is a WAIS-IV subtest (reported as NART50 in Table 1 and available with the MRI data).

**Figure 6:**
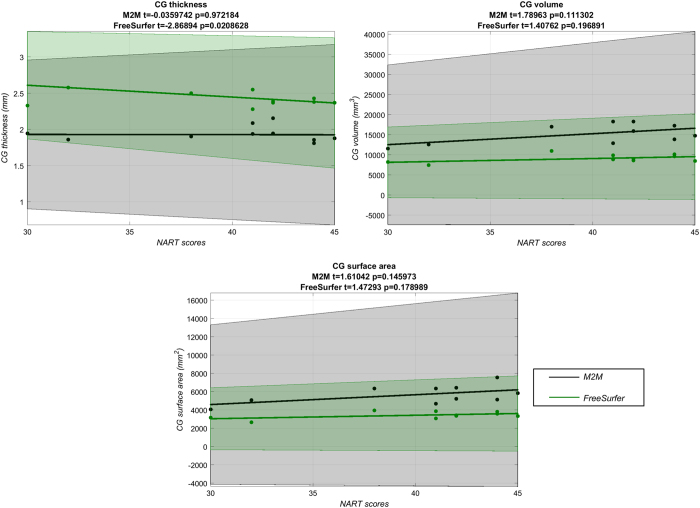
Regression analysis on CG metrics. CG thickness (**a**), volume (**b**), and surface area (**c**) as measured by M2M and FreeSurfer with respect to NART scores.

The highest density interval (HDI) of the difference in regression coefficients did not differ between our parcellation and FreeSurfer’s for CG thickness (HDI: [−0.001 0.0006], Fig. 6a), but was statistically different for both CG volume (HDI: [−0.0016 −0.0021], Fig. 6b) and surface area (HDI: [−0.0025 −0.0002], Fig. 6c), with no association using either parcellation scheme.

It is understandable that when dealing with large datasets it is not possible to manually segment the entire ground truth. However, from what we have observed with our small cohort, cortical variability is not rare. It is therefore crucial to be aware of the variability details of any regions of interest, and when working with automated tools to check for variability considerations to best assess the implications this may have on the results, if any. The data presented here provide in that sense a good testing ground for automated MRI parcellation.

## Usage Notes

All previously described data is freely available at the Edinburgh DataShare repository (Data Citation 1) under the CCBY license.

Masks2Metrics is a tool that is freely available on GitHub, at https://github.com/Edinburgh-Imaging/Masks2Metrics, under the GNU General Public License (archived version used for the results presented available at Edinburgh DataShare repository^27^). The tool has also been published in the Journal of Open Source Software (JOSS)^28^.

Results for the Technical Validation section were derived by running our Matlab code (Matlab_code_to_derive_stats_and_figs.m, Data Citation 2) which uses the parcel metrics of Data Citation 2 (SuperiorFrontalGyrus.tsv, SupraMarginalGyrus.tsv, and CingulateGyrus.tsv).

## Additional information

**How to cite this article**: Mikhael, S.S. *et al*. Manually-parcellated gyral data accounting for all known anatomical variability. *Sci. Data*. 6:190001 https://doi.org/10.1038/sdata.2019.1 (2019).

**Publisher’s note**: Springer Nature remains neutral with regard to jurisdictional claims in published maps and institutional affiliations.

## Supplementary Material



## Figures and Tables

**Table 1:** Demographics and cognitive scores of the 10 subjects used for this study, with scores reported in the order in which the tests were administered.

**Subject ID**	1	2	3	4	5	6	7	8	9	10
**Subject Demographics**										
Age	57	56	63	64	64	57	59	61	62	55
Gender	M	M	F	F	M	F	F	F	M	M
**Cognitive Scores**										
LogMemOne25	20	18	15	19	15	18	20	22	19	16
NART50	44	38	32	30	41	42	45	42	41	44
DigSymb133	79	79	57	60	69	77	74	82	62	72
BlockDes68	62	54	42	31	59	44	45	45	32	51
LettNumSeq21	14	16	11	14	10	13	19	10	10	10
SimpleRTMean	0.221	0.225	0.233	0.229	0.241	0.223	0.231	0.229	0.233	0.262
SimpleRTSD	0.028	0.031	0.024	0.064	0.03	0.033	0.038	0.047	0.031	0.044
FourChRTMean	0.612	0.529	0.617	0.556	0.595	0.563	0.481	0.546	0.585	0.602
FourChRTSD	0.124	0.096	0.155	0.117	0.103	0.103	0.091	0.111	0.113	0.11
FourChNoErr	1	0	0	0	1	0	0	2	0	2
FourChRTErr	0.41	0	0	0	0.508	0	0	0.556	0	0.633
FourChSDErr	0	0	0	0	0	0	0	0.214	0	0.035
MatrReasg26	21	21	18	19	17	20	14	23	10	17
VerbFluC	16	18	11	11	15	17	19	18	14	13
VerbFluF	10	14	13	13	15	15	18	17	12	20
VerbFluL	16	14	17	13	12	12	17	18	14	17
LogMemTwo25	17	18	13	14	12	19	17	17	17	10
VFtot	42	46	41	37	42	44	54	53	40	50
LogMemOne25: Logical Memory 1; NART50: National Adult Reading Test; DigSymb133: Digit-Symbol Substitution Test; BlockDes68: Block Design Test; LettNumSeq21: Letter Number Sequencing; Both the Simple and Four-Choice Reaction Time Tests^31^ were administered, reporting overall mean (SimpleRTMean and FourChRTMean, respectively), and overall standard deviation for correct responses (SimpleRTSD and FourChRTSD, respectively); Additionally, the number of errors (FourChNoErr) as well as the mean (FourChRTErr) and standard deviation (FourChSDErr) of response time for incorrect responses were reported for the Four-Choice Reaction Time Test. MatrReasg26: Matrix Reasoning test; VerbFluC/F/L: Verbal Fluency test for the letters ‘C’, ‘F’, and ‘L’, respectively; LogMemTwo25: Logical Memory 2; VFtot: total score for Verbal Fluency test (letters ‘C’, ‘F’ and ‘L’).										

**Table 2:** A summary of the T1w MRI parameters.

**T1w MRI parameters**	
*Scanning sequence*	Fast spoiled gradient-echo
*Repetition time (TR)*	9.8 ms
*Echo time (TE)*	4.01 ms
*Flip angle*	8°
*Inversion time*	500 ms
*Matrix size*	256 × 156 × 256
*Voxel size*	1 mm × 1.3 mm × 1 mm

**Table 3:** A summary of the T2w MRI parameters.

**T2w MRI parameters**	
*Scanning Sequence*	Spin echo
*Repetition time (TR)*	11320 ms
*Echo Time (TE)*	104.9 ms
*Flip Angle*	90°
*Matrix size*	256 × 256 × 80
*Voxel size*	1 mm × 1 mm × 2 mm

**Table 4:** A summary of anatomical variations observed in our dataset.

Subject ID	Hemisphere	SFG	SMG	CG
**1**	*left*	2D: 1 in CS, 3 in SFS 3D: 1 in CS	2D: 1	2D: 4 in CS 3D: 1 in CS Double CG
	*right*	2D: 1 in CS, 2 in SFS	2D: 2	2D: 5 in CS
**2**	*left*	2D: 1 in CS, 3 in SFS	2D: 1	2D: 6 in CS 3D: 1 in CS
	*right*	2D: 2 in CS, 2 in SFS	2D: 2	2D: 8 in CS
**3**	*left*	2D: 1 in CS, 3 in SFS	2D: 0	2D: 5 in CS
	*right*	2D: 1 in CS, 4 in SFS	2D: 1	2D: 6 in CS
**4**	*left*	2D: 0 in CS, 2 in SFS	2D: 1	2D: 3 in CS
	*right*	2D: 1 in CS, 1 in SFS	2D: 1	2D: 5 in CS
**5**	*left*	2D: 2 in CS, 1 in SFS 3D: 2 in CS	2D: 0	2D: 11 in CS 3D: 2 in CS Double CG
	*right*	2D: 1 in CS, 4 in SFS	2D: 2	2D: 5 in CS
**6**	*left*	2D: 1 in CS, 4 in SFS 3D: 1 in CS	2D: 0	2D: 8 in CS Double CG
	*right*	2D: 1 in CS, 3 in SFS	2D: 2	2D: 8 in CS Double CG
**7**	*left*	2D: 1 in CS, 3 in SFS	2D: 2	2D: 3 in CS
	*right*	2D: 2 in CS, 1 in SFS 3D: 1 in CS	2D: 0	2D: 3 in CS
**8**	*left*	2D: 1 in CS, 2 in SFS	2D: 2	2D: 8 in CS 3D: 1 in CS Double CG
	*right*	2D: 0 in CS, 1 in SFS	2D: 4	2D: 4 in CS
**9**	*left*	2D: 1 in CS, 1 in SFS	2D: 0	2D: 2 in CS 3D: 1 in CS
	*right*	2D: 0 in CS, 3 in SFS	2D: 0	2D: 5 in CS 3D: 1 in CS
**10**	*left*	2D: 0 in CS, 3 in SFS	2D: 2	2D: 3 in CS 3D: 1 in CS
	*right*	2D: 0 in CS, 2 in SFS	2D: 1	2D: 4 in CS Double CG
The variations include double sulcal and gyral occurrences (reported as ‘double CG’), sulcal (in the SFG and CG) and gyral (in the SMG) discontinuities as observed in 2-dimensional space (2D, looking at slices in the direction along which the gyri were drawn, i.e., axial for the SFG and sagittal for the SMG and CG) as well as in 3-dimensionsal space (3D). CS: Cingulate sulcus; SFS: superior frontal sulcus.				

**Table 5:** The number of paired WM and GM segments varied across subjects and hemispheres depending on cortical variability and the degree of folding.

**ROI details- # paired WM and GM segments**										
*Subject ID*	1	2	3	4	5	6	7	8	9	10
*SFG (L/R)*	52/55	58/52	59/53	51/40	24/39	30/39	46/58	15/32	37/57	37/31
*SMG (L/R)*	11/9	11/16	11/10	15/6	8/9	8/8	9/4	10/11	3/7	7/7
*CG (L/R)*	30/23	31/41	16/23	20/30	54/27	39/30	24/25	34/33	27/35	27/27

**Table 6:** Gyral metrics, averaged for the left and right hemispheres, as measured by M2M, FreeSurfer (FS), and mean modified Hausdorff distance (MMHD).

Method	Metric	SFG	SMG	CG
**M2M**	*Thickness (mm)*	2.48[2.22 2.65]	2.28[1.91 2.44]	1.92[1.82 2.02]
	*Volume (mm*^*3*^)	20178.41[11612.83 22082.02]	3694.25[2318.42 5645.28]	15355.40[11744.02 17526.65]
	*Surface area (mm*^*2*^)	5225.54[2460.34 5812.78]	1061.21[660.83 1558.56]	5627.03[4070.78 6370.85]
**FS**	*Thickness (mm)*	2.50[2.33 2.54]	2.46[2.31 2.53]	2.40[2.29 2.51]
	*Volume (mm*^*3*^)	19545.81[15159.22 20869.87]	10282.99[8050.29 11112.30]	9051.20[7468.71 9978.39]
	*Surface area (mm*^*2*^)	6605.53[5116.19 6997.09]	3660.12[3076.91 3876.87]	3433.69[2730.04 3784.79]
**MMHD**	*Thickness (mm)*	2.64[2.45 2.79]	2.78[2.33 2.94]	2.70[2.47 2.92]
Median and 95% highest density intervals (median [HDI]) are reported for the superior frontal gyrus (SFG), supramarginal gyrus (SMG) and cingulate gyrus (CG). Raw (source by hemisphere and subjects) data are available under Data Citation 2 (SuperiorFrontalGyrus.tsv, SupraMarginalGyrus.tsv, and CingulateGyrus.tsv).				
